# Supplementary material for: Advances in Mass Rearing Pseudophilothrips ichini (Hood) (Thysanoptera: Phlaeothripidae), a Biological Control Agent for Brazilian Peppertree in Florida
Source: Insects. 2021 Sep 3;12(9):790. doi: 10.3390/insects12090790 (PMC8468526; doi:10.3390/insects12090790)
Supplement: Supplementary file 1 [file insects-12-00790-s001.zip › insects-1331341-supplementary.pdf]

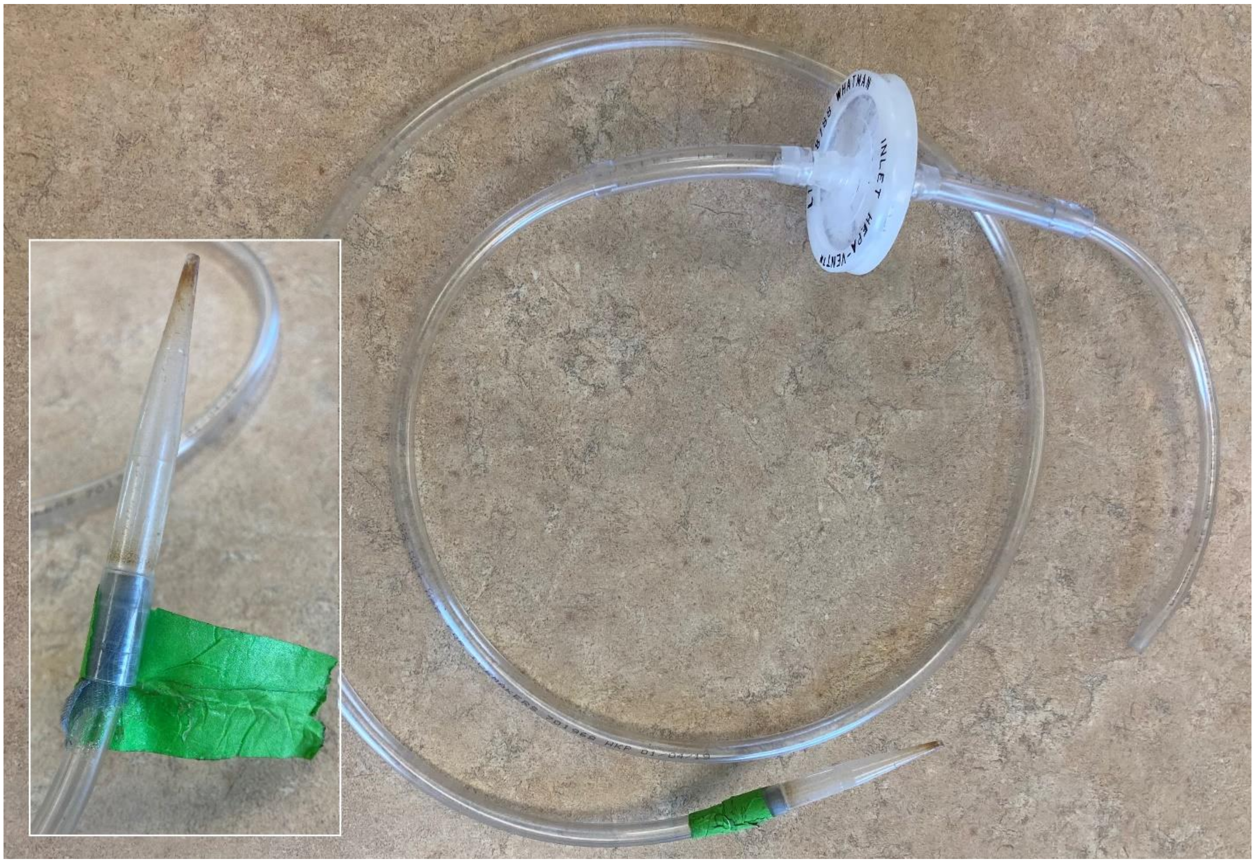

Figure S1. Custom-built manual aspirator used to collect adult thrips to transfer to new rearing cages. A Whatman® in-line HEPA filter (cat. # 6723-500; GE Healthcare Life Sciences) was added to a roughly 1 m section of 6.35 mm (¼ in) outer diameter vinyl airline tubing. Two roughly 3 cm sections of 6.35 mm (¼ in) inner diameter airline tubing made the connection to the barbs on the filter. The inset photo shows the assembly of the thrips collection chamber, which consisted of a 1 × 1 cm piece of 250 μm fine mesh placed over the end of the 6.35 mm inner diameter vinyl tubing and the wide end of a 1 mL autopipette tip (the chamber itself) fit snugly over it. A piece of tape was used to hold the assembly together and make an air-tight seal. The small end of the chamber was cut to have a 1.5–2 mm opening. This diameter provided the ideal balance between high-velocity inflow and minimizing damage to thrips while passing through the narrow opening. We typically aspirated groups of 50 to at most 100 adult thrips at a time. Thrips were expelled from the chamber by pointing the small end down and synchronously tapping the side of the chamber with a pencil while exhaling brief but sharp puffs of air.
